# Reconstructing the origin and transmission dynamics of the 1967–68 foot-and-mouth disease epidemic in the United Kingdom^☆^

**DOI:** 10.1016/j.meegid.2013.09.009

**Published:** 2013-12

**Authors:** Caroline F. Wright, Nick J. Knowles, Antonello Di Nardo, David J. Paton, Daniel T. Haydon, Donald P. King

**Affiliations:** a The Pirbright Institute, Ash Road, Pirbright, Woking GU24 0NF, UK; b Institute of Biodiversity, Animal Health and Comparative Medicine, College of Medical, Veterinary and Life Sciences, University of Glasgow, Glasgow G12 8QQ, UK

**Keywords:** Foot-and-mouth disease, Epidemic, Full-genome sequencing, Phylogenetics, United Kingdom

## Abstract

A large epidemic of foot-and-mouth disease (FMD) occurred in the United Kingdom (UK) over a seven month period in Northwest England from late 1967 to the summer of 1968. This was preceded by a number of smaller FMD outbreaks in the country, two in 1967, in Hampshire and Warwickshire and one in Northumberland during 1966. The causative agent of all four events was identified as FMD virus (FMDV) serotype O and the source of the large epidemic was attributed to infected bone marrow in lamb products imported from Argentina. However, the diagnostic tools available at the time were unable to entirely rule out connections with the earlier UK FMD outbreaks, as well as other potential sources from Europe. The aim of this study was to apply molecular sequencing to investigate the likely source of this epidemic using VP1 region and full genome (FG) sequences determined directly from clinical epithelium samples (*n* = 13) or cell culture isolates (*n* = 6), from this and contemporary outbreaks in the UK, Europe and South America. Analysis of the VP1 sequences provided evidence for at least three separate incursions of FMDV into the UK including one independent introduction that was responsible for the main 1967/68 epidemic. Analysis of FG sequences from the main 1967/68 outbreak (*n* = 10) revealed nucleotide substitutions at 94 genomic sites providing evidence for the linear accumulation of nucleotide substitutions (rate = 2.42 × 10^−^^5^ nt substitutions/site/day). However, there were five samples where this linear relationship was absent, indicating evolutional dormancy of the virus*,* presumably outside a host. These results help define the evolutionary dynamics of FMDV during an epidemic and contribute to the knowledge and understanding from which to base future outbreak control strategies.

## Highlights

• Study characterised genomic sequences from archived FMDV samples collected during the 1960s.
• The epidemic in 1967–68 occurred as a result of an independent introduction of FMDV into the UK.
• Molecular clock used to identify viral sequences that accrued fewer than expected substitutions.
• Study supports the use of sequences to trace RNA viruses during outbreaks and epidemics.

## 1 Introduction

Foot-and-mouth disease (FMD) is associated with severe productivity losses in cloven-hoofed animals, characterized by vesicular lesions of the feet tongue, snout and teats as well as fever and lameness (Arzt et al., 2011). The etiological agent, FMD virus (FMDV), is a member of the genus *Aphthovirus* in the family *Picornaviridae*. Serotype O, which can be sub-divided into 11 geographically distinct genetic topotypes (Samuel and Knowles, 2001), is the most prevalent of the seven serotypes of FMDV. High mutation rates of FMDV RNA polymerase (10^−^^3^–10^−^^5^ per nt per transcription cycle), coupled with large population sizes and a rapid replication rate, results in the fast evolution of this virus within infected hosts (Domingo et al., 2003). Although predominantly spread by direct or indirect contact with infected animals, their secretions or associated products, FMDV can also be disseminated over extensive distances by air and windborne routes or via inanimate fomites, causing incursions in areas previously free of the disease (Arzt et al., 2011).

FMD was first recorded in the United Kingdom (UK) in 1839 but has not been endemic in the UK since 1884 (M.A.F.F., 1965). However, outbreaks occurred almost every year until 1968, with virus introductions attributed to spread from Europe and South America. A major FMD epidemic, starting in October 1967 in Shropshire, UK, caused 2364 outbreaks, on 2346 farms, 18 of which were infected twice (M.A.F.F., 1965). The epidemic was controlled after seven months using a stamping-out policy combined with movement restrictions imposed upon susceptible animals and the last outbreak was reported on the 4th of June 1968. Although cases occurred in North and South Wales, Lancashire, Westmorland, Derbyshire, South-West Midlands, and the East Midlands, the vast majority of the affected farms (2228) were located in the North-West Midlands, which had the highest density of dairy cattle in the country at the time. Apart from two years (1963 and 1964), FMD had sporadically been present in the UK on all thirteen years preceding this epidemic, including three sets of outbreaks affecting Northumberland (32 outbreaks over approximately three months, 1966), Hampshire (29 outbreaks during January, 1967) (Sellers and Forman, 1973) and Warwickshire (5 outbreaks over 3 days in September, 1967) (Sellers and Forman, 1973).

The UK Government commissioned the Northumberland Report (Anon, 1969a) that made four principal conclusions regarding the primary source and initial spread of the epidemic. First, that it was not possible to categorically establish the origin of the 1967/68 epidemic. Second, that the most probable source of the epidemic was infected meat imported from South America. Third, that, as the Ministry of Agriculture had also attributed an earlier outbreak in 1967 in Warwickshire to South American meat, this event remained as a potential link to the main 1967/68 epidemic. Finally, that it was difficult to explain the epidemic’s rapid development and extension other than by accepting that a number of foci were established almost simultaneously.

Since 1968, FMDV diagnostic tools have advanced from serologically based tests, such as the complement fixation and virus neutralization tests, to molecular sequencing of viral RNA. Determination of the genetic sequence for one of three surface exposed capsid proteins of FMDV (VP1) and the full genome (FG), has not only enabled the global tracing of FMD transmission but provided the resolution at which disease spread can be monitored at the epidemic scale (Abdul-Hamid et al., 2011a; Cottam et al., 2008a; Knowles et al., 2005; Samuel and Knowles, 2001; Valdazo-Gonzalez et al., 2012). As well as providing the genetic profile of an epidemic, FG sequencing of FMDV can yield insights into the processes shaping this profile, for example, by testing for the presence of a molecular clock in terms of nucleotide (nt) substitution rate. Previous studies have demonstrated that nt changes, from the earliest FMDV sample, accrue in a linear ‘clock-like’ way with time (Villaverde et al., 1991b; Elena et al., 1992; Haydon et al., 2001; Cottam et al., 2006; Valdazo-Gonzalez et al., 2012), resulting from continuous viral replication within susceptible hosts. The aim of this study was to apply current molecular sequencing tools to characterize the 1967/68 FMD epidemic by establishing its relationship with contemporary UK outbreaks, determining whether this epidemic was mono or polyphyletic and measuring the rate of mutation accumulation over time. Subsequent phylogenetic analysis will help to clarify some of the issues highlighted by the Northumberland Report (Anon, 1969a,b), expanding the knowledge fed into future disease control programmes.

## 2 Material & methods

### 2.1 Samples

This study accessed archived vesicular epithelium samples (*n* = 13) from the World Reference Laboratory for FMD (WRLFMD) at The Pirbright Institute, which had been stored at −20 °C in 0.04 phosphate buffer (M25; disodium hydrogen phosphate, potassium dihydrogen phosphate, pH 7.5) and 50% (vol/vol) glycerol. The 13 clinical samples were collected from early in the Northumberland outbreak (OB-Northumb), the beginning of the Hampshire outbreak (OB-Hants), the beginning of the Warwickshire outbreak (OB-Warks), and a total of ten samples collected approximately every month from near the beginning (the isolate from the index case on Bryn Farm was not available), to the end of the 1967/68 outbreak. As the index case for the 1967/68 outbreak occurred in Shropshire, this outbreak was designated as the Shropshire outbreak (OB-Shrops). All 13 samples had previously been found FMDV positive by the complement fixation test on original submission to the WRLFMD at the time of these outbreaks. All clinical samples from which FG sequences were determined (*n* = 12) are detailed in Table 1. Additionally, FMDV VP1 sequences from contemporary outbreaks in the UK, Europe and South America were determined (*n* = 12), and comprised of those determined from a single epithelium sample from OB-Northumb and cell culture isolates (*n* = 6), as well as those available from GenBank (*n* = 5), as detailed in Table 2. Finally, VP1 sequences determined from isolates collected from outbreaks in France and the UK in 1981 (*n* = 3) were also included for comparative purposes (Table 2).

### 2.2 Sample preparation and RNA extraction

RNA was extracted from epithelium suspension preparations as previously described (Cottam et al., 2006). Briefly, a 10% tissue suspension was prepared with a pestle and mortar in a class II safety cabinet using 0.04 M phosphate buffer and approximately 1.5 g of each of the vesicular epithelium samples. The suspension was then centrifuged for 10 min at 3500*g* at room temperature and the supernatant removed to be stored at −80 °C until tested. Total RNA was extracted (TRIzol, Invitrogen, Paisley, UK) from all epithelium suspensions before reverse transcription and amplification by PCR. Total RNA was extracted from 460 μl cell culture supernatant by using RNeasy kits (Qiagen Ltd., Crawley, West Sussex, UK), according to the manufacturer’s instructions, resuspended in 50 μl nuclease-free water and stored at −80 °C.

### 2.3 RT-PCR and DNA sequencing

The following reverse transcription method was modified from that previously described (Cottam et al., 2006). Briefly, extracted RNA (15 μl) was added to 3 μl 10 mM oligo-dT primer UKFMD/Rev6 (Cottam et al., 2006), 3 μl 10 mM deoxynucleoside triphosphate mix and then incubated at 70 °C for 3 min followed by 4 °C for 3 min. Nineteen microlitres of freshly prepared RT mix (8 μl 5× RT buffer [Invitrogen], 2 μl 0.1 mM dithiothreitol, 2 μl RNase OUT [Invitrogen], 5 μl nuclease-free water) was added to the sample followed by 2 μl of an enzyme with high fidelity (Superscript III reverse transcriptase, Invitrogen). The sample was then incubated at 45 °C for 60 min, after which the cDNA synthesis reaction was terminated by incubation at 85 °C for 5 min. The cDNA was then cleaned using QIAquick PCR purification kits (QIAGEN), eluted in 40 μl of nuclease-free water before storage at −20 °C.

Amplification by RT-PCR of the VP1 encoding region was achieved using the primer sets previous described (Abdul-Hamid et al., 2011b; Knowles et al., 2005). The protocol used for FG PCR amplification was modified from that previously described (Cottam et al., 2008b). Briefly, twenty-three overlapping PCR fragments covering the FMDV genome were amplified by adding 3 μl of each cDNA to 47 μl of master mix (5 μl 10× buffer, 2 μl MgSO_4_, 1 μl 10 mM deoxynucleoside triphosphate mix, 1 μl 10 mM forward primer, 1 μl 10 mM reverse primer, 0.25 μl Platinum Taq DNA Polymerase Hi-Fidelity [Invitrogen], 37 μl nuclease-free water). Details of the RT and PCR primers used are as previously published (Cottam et al., 2008b). Samples were run on a PCR program cycle of initial denaturation at 94 °C for 5 min and then 39 cycles of 94 °C for 30 s, 55 °C for 30 s, and 72 °C for 1 min, ending with incubation at 72 °C for 7 min. PCR products were cleaned up using QIAquick PCR purification kits (Qiagen), eluting in 50 μl of nuclease-free water. In order to visualize amplified DNA to check quality and specificity of the product, 3 μl was run on a 1% agarose gel at 100 V for 35 min alongside a quantitative ladder (GeneRuler 100 bp LadderPlus, MBI Fermentas). Sequencing reactions were performed using the Applied Biosystems BigDye Terminator V3.1 Cycle Sequencing Kit and an ABI 3730 Genetic Analyser.

### 2.4 Sequence analysis

The raw sequence data were assembled using SeqMan Pro™ 10.1.1 (DNASTAR, Inc.) followed by BioEdit 7.1.11 (Hall, 1999) for all subsequent sequence manipulations and nt difference counts between sequences. Before performing the phylogenetic reconstruction, MEGA 5.2 (Tamura et al., 2011) was employed to determine the best fitting nucleotide substitution model by Bayesian information criterion (BIC) (Posada and Buckley, 2004). The evolutionary history of all VP1 sequences was then inferred computing the maximum likelihood (ML) tree in MEGA 5.2 using the Kimura 2-parameter + Γ5 model of base substitution (Kimura, 1980). The genealogical network underlying the relationship between all 12 FG sequences examined was computed based on statistical parsimony implemented in TCS 1.21 (Clement et al., 2000). In order to include a candidate most likely common ancestor in the TCS analysis, a FASTA search of all publically available FG sequences was completed using the FG sequences for OB-Shrops and the top six hits included in the TCS analysis. Putative recombinant sequences were identified using Simplot 3.5.1 (Lole et al., 1999) setting a sliding window 200 bp wide with a step size of 20 bp. Results obtained were then confirmed by bootscan analysis of 1000 bootstrapped trees generated using the Kimura 2-parameter model (Kimura, 1980).

In order to compare the rate of nt substitution observed during OB-Shrops and a more recent UK FMD outbreak of equivalent size and duration, a molecular clock was fitted to the first five sequences from OB-Shrops (A–E) and 42 sequences collected during the UK 2001 FMD outbreak and previously analysed (Cottam et al., 2006, 2008a; Konig et al., 2009). Markov chain Monte Carlo techniques were implemented in the software package BEAST 1.7.5 (Drummond et al., 2012), where a random local clock rate of evolution (Drummond and Suchard, 2010), no prior assumption of population size (Bayesian Skyline plot), and the HKY85 + Γ4 (Hasegawa et al., 1985) model of base substitution with empirical base frequencies was assumed. Once extracted from BEAST, the difference in clock rate observed during OB-Shrops and the 2001 UK outbreak was tested using the Student *t*-test with Welch’s approximation (Welch, 1947) in R 3.0.1 (R Core Team, 2013).

## 3 Results

### 3.1 VP1 sequence analysis

**Fig. 1:**
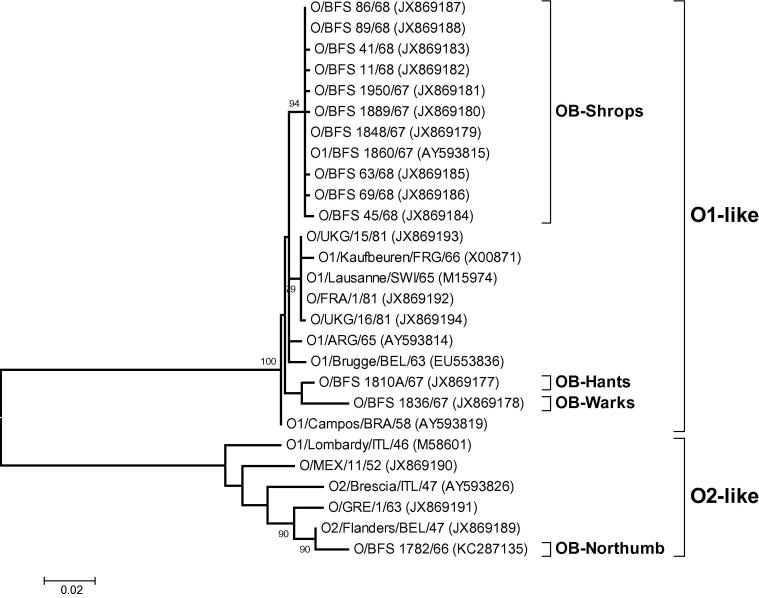
Unrooted Maximum Likelihood tree showing the relationships between 27 VP1 sequences for FMDV isolates collected from the UK and other contemporary outbreaks in Europe and South America. The percentage of replicate trees in which associated sequences clustered together in the bootstrap test (1000 replicates) are shown next to the branches (>70%).

VP1 sequence analysis was used to define the genetic relationships between OB-Shrops, previous FMD outbreaks in the UK, as well as contemporary outbreaks in Europe and South America. Fig. 1 clearly shows that sequences determined from the four UK FMD outbreaks (OB-Shrops, OB-Warks, OB-Hants and OB-Northumb) were on three separate phylogenetic lineages, with the OB-Hants and OB-Warks samples found to be more closely related to each other than to any other isolate analysed here (bootstrap values > 70%). Both of these trees indicated that the OB-Northumb sample was more closely related to earlier outbreaks in Europe (Belgium and Greece) and part of a lineage (O_2_) that has not been reported to have been present in South America. Furthermore, samples taken from the beginning and end of OB-Shrops were shown to be more closely related to South American isolates compared to the samples from previous UK FMD outbreaks (OB-Shrops ‘A’ and ‘J’ both demonstrated a 12 and 19 nt difference from OB-Hants and OB-Warks respectively, whereas OB-Shrops ‘A’ demonstrated a 8 nt difference and OB-Shrops ‘J’ a 6 nt difference from O_1_/Campos/BRA/58 [AY593819]).

Interestingly, no nt differences were observed within the VP1 sequence between samples ‘A’ and ‘J’ taken at the beginning and the end of OB-Shrops, although the samples were taken 217 days apart and a total of only 7 nt differences were observed when VP1 sequences were compared within all of the samples collected from the ten OB-Shrops premises). The branch containing VP1 sequences from early and late samples of OB-Shrops also contained the VP1 sequence that had been previously determined for an isolate (BFS 1860; GenBank accession AY593815) from this outbreak (see Section 3.3 for further details and FG sequence analysis of this isolate).

### 3.2 Full genome sequence analysis between outbreaks

The assembled FG sequences of the 12 epithelium samples analysed were unique and ranged in length between 8183 nt (OB-Shrops, Sample E, which contained a single deletion of 3 nt discussed Section 3.3), 8187 nt (single sample for both OB-Hants and OB-Warks, which both contained a single inserted ‘A’ nt immediately after position 8145), and 8186 nt for the remaining nine samples. No ambiguities were found within any of the VP1 or FG sequences. For analyses, primer derived sequences (<0.4% of the total genome length), were omitted at the 3′ and 5′ ends of the genome (15 and 8 nt respectively), as well as at the 3′ and 5′ ends of the poly(C) tract (5 and 4 nt respectively). The poly(C) and poly(A)tracts of these viruses were not directly sequenced in this study and were substituted by artificial sequences in these regions of 10 nt long C and A repeats, respectively. The poly(C) tract within the FMDV genome can vary between 100 and 420 nt in length and the poly(A) tail between 35 and 100 nt (Carrillo et al., 2005).

**Fig. 2:**
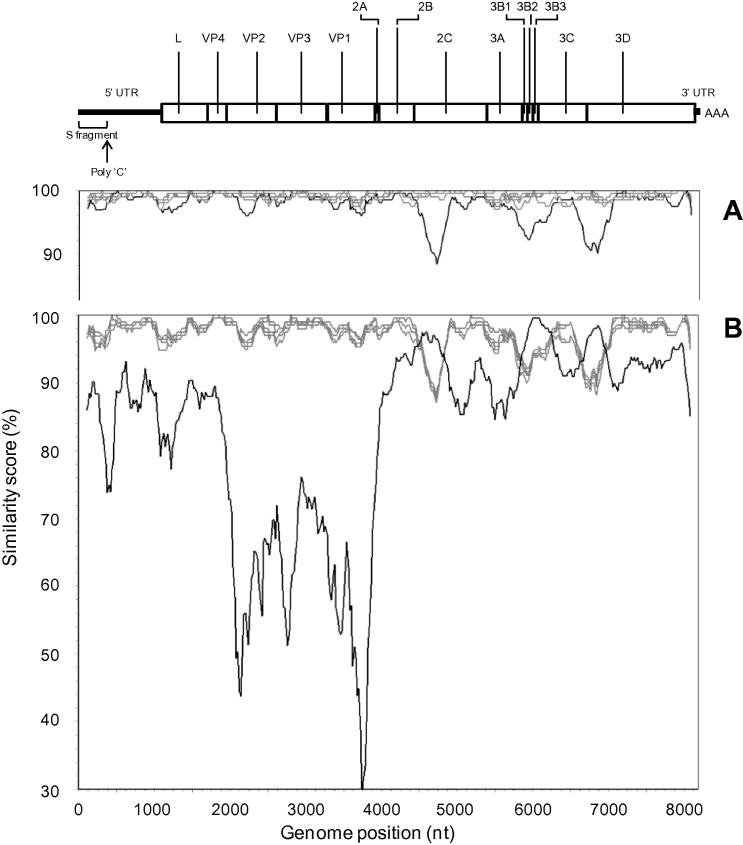
Recombination analysis using SimPlot. (A) all FG sequences from OB-Shrops (grey trace), OB-Hants (grey trace) and OB-Warks (black trace) queried against O_1_/Campos/BRA/58 (AY593819). (B) all previous FG sequences analysed (gray traces) plus that of A_26_/Argentina/66 (AY593770) (black trace) queried against the FG sequence of the OB-Warks sample. Analysis performed using a sliding window size of 200 nt moving in steps of 20 nt along the alignment. The pairwise similarity values were plotted at the midpoint of the 200 nt window. At the top of the figure, a fully annotated FMDV FG sequence is represented.

The FG sequence from OB-Warks and OB-Hants were different to the FG sequences from OB-Shrops: differing by 232-255 nt for OB-Warks and 102–125 nt for OB-Hants. Of the samples analysed, OB-Warks was most closely related to OB-Hants although the genetic difference between the two samples was still substantial (difference of 214 nt). In order to establish the reliability of these inferred phylogenetic relationships, recombination analysis was performed. Fig. 2 (A) identified three areas where the OB-Warks sequence identity was lower (from the 5′ end of P2 (2C) to the P3 region (3D) at nt positions 4600–4900; 5800–6200 and 6600–7000, respectively), highlighting regions of increased genome divergence compared to all the other reference sequences when queried against the FG sequence, O_1_/Campos/BRA/58[AY593819]. These fragments (∼300–400 nt) from the OB-Warks sequence, spanning each of these areas, were submitted for FASTA analysis (http://www.ebi.ac.uk/Tools/sss/fasta/nucleotide.html): the closest match was a sequence from A_26_/Argentina/66 (AY593770) (97-98% nt identity). Fig. 2B shows the subsequent similarity plot (specifications as described above) of the OB-Warks sequence queried against all other determined FG sequences and A_26_/Argentina/66. Regions of highest similarity between OB-Warks and A_26_/Argentina/66 are clearly correlated with regions of lowest similarity between all other reference sequences and the OB-Warks sequence. These results are indicative of recombination events having occurred between an ancestor of OB-Warks and a virus closely related to A_26_/Argentina/66 presumably via coinfection of host cells.

### 3.3 Full genome sequence analysis within an outbreak

Using the earliest sequence determined from OB-Shrops (sample A) as a reference, the remaining nine FG sequences of this outbreak were found to contain 94 nt substitutions across the genome. Within the coding region, these nt changes were mainly synonymous (*n* = 65), the majority of which were transitions (*n* = 62), compared to non-synonymous mutations (*n* = 17). Eleven mutations were found within the 5′ UTR and one was found within the 3′ UTR. In addition, a single deletion of three nt (ACC at positions 190 to 192, with respect to GenBank sequence JX869183) was found within the 5′ UTR of a single sample (sample E). The single stem-loop secondary structure previously predicted for the FMDV S-fragment RNA sequence (Clarke et al., 1987; Newton et al., 1985), was tested using RNAStructure 5.4 (Lu et al., 2009), and found to be maintained, with small conformational adjustments, despite this deletion. While the loop apex of this stem-loop structure maintained an A–C–C–U–C conformation within the sequence for sample E, the adjacent stem was elongated by two base pairs and the following two loops were smaller by one and six nt respectively (data not shown). However, the nt composition of the predicted loop apex was not conserved on examination of approximately 100 FMDV S-fragment sequences (representing all seven FMDV serotypes).

**Fig. 3:**
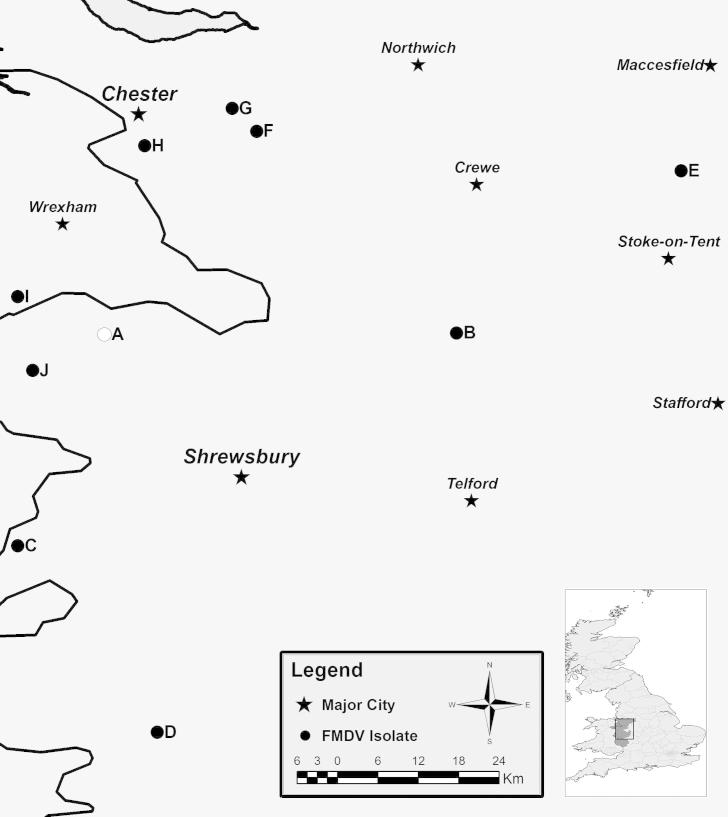
Geographical locations of the premises (●) for all the ten samples from OB-Shrops. White circle represents the farm designated as the index case and stars show major towns in the region. Solid black line at the left margin of the figure represents the English/Welsh border.

**Fig. 4:**
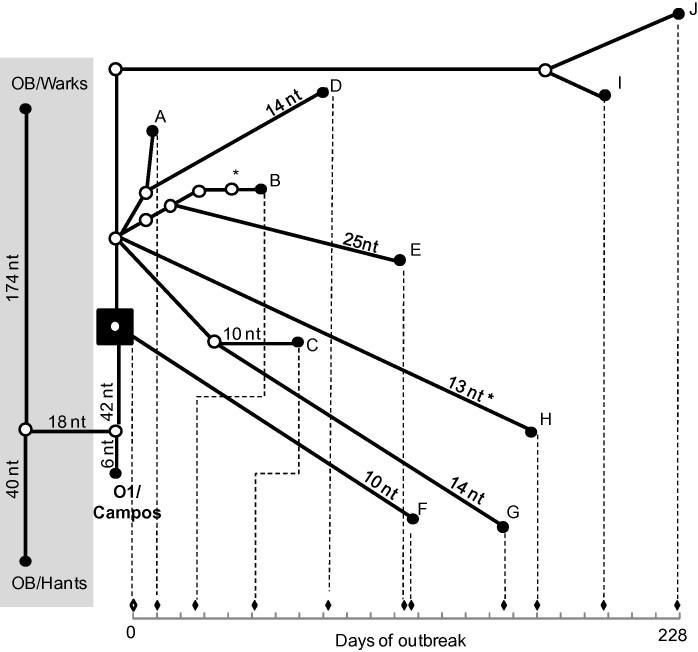
Statistical parsimony analysis by TCS. Ten FG sequences (A–J) derived from clinical epithelium samples collected during OB-Shrops are shown in relation to those from OB-Hants and OB-Warks (past UK outbreaks highlighted in gray box) as well as the most similar South American sequence (O_1_/Campos/BRA/58 [AY593819]). The estimated most likely common ancestor is highlighted within a black box. Unless otherwise stated, each connecting branch line represents a single nt substitution, with each dot representing a putative ancestor virus. Node tips for all OB-Shrops sequences are correlated to an outbreak timeline so that branch length is proportional to time. (∗) nt substitution occurred twice on independent branches.

The maximum genetic difference seen between samples studied from OB-Shrops was of 42 nt (E–G), with a minimum of 2 nt (I–J). The geographical map of all OB-Shrops samples locations is provided in Fig. 3. The genealogy network of all ten samples from this outbreak, in relation to OB-Hants and OB-Warks samples plus the most likely common ancestor using O_1_/Campos/BRA/58 (AY593819), implemented by TCS is shown in Fig. 4. The most likely common ancestor, as estimated by statistical parsimony analysis, was maintained after addition of all publically available FG sequences for the 1967/68 outbreak (EU448370, EU448369, EU448368, AY593816, AY593815 [data not included in Fig. 4]). All publically available FG sequences were from cell culture passaged viruses (passage numbers unknown), originally derived from a single epithelium sample (collected on the 1st of November 1967 in Wrexham), which was designated serotype O_1_ British Field Sample 1860 (O_1_ BFS 1860) and used as the type virus for this outbreak. It should be noted that, although additional nt substitutions may have been introduced into the viral genome of cell culture passaged isolates, these do not change the overall conclusions of this analysis.

**Fig. 5:**
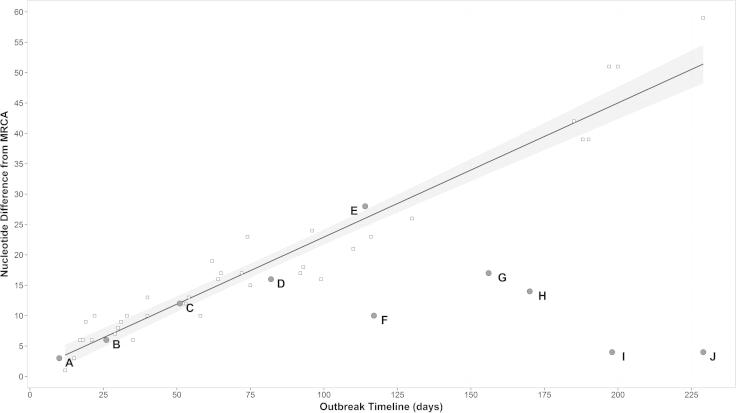
Accumulation of nt substitutions over time during OB-Shrops. FG sequences derived from samples A–J are included (grey dots). The regression line with corresponding 95% confidence intervals was fitted for 42 FG sequences that are available from the 2001 epidemic in the UK (white squares).

Longer branch lengths in Fig. 4 indicate a relatively slow accumulation of nt substitutions (observed in samples F–J), whereas shorter branch lengths indicate a relatively fast accumulation of nt substitutions (observed in samples A–E). Fig. 5 shows the accumulation of nt substitutions from the most likely common ancestor (estimated by TCS analysis), for the ten OB-Shrops samples against time. The estimated most likely common ancestor was dated as the 21st of October 1967, according to the date on which FMD symptoms were first reported on Bryn Farm (Anon, 1969a). Fitting a linear regression model for 42 sequences previously generated from the UK 2001 epidemic (also involving a serotype O virus) provided evidence that sequences for samples A–E were shaped following the same linear accumulation of nt substitution over time, whilst this relationship was absent for sequences for isolates F–J (Fig.5). In addition, slope values calculated for the UK 2001 samples (*β* = 0.225, *R*^2^ = 0.94) and the isolates A–E (*β* = 0.229, *R*^2^ = 0.97) were almost identical, as opposed to the parameter returned for the isolates F–J (*β* = -0.086, *R*^2^ = 0.38). No statistical difference was observed between the molecular clock rate (expressed as nt substitutions/site/year) estimated using BEAST for samples A–E from OB-Shrops (8.74 × 10^−^^3^, 95%HPD 8.73 × 10^−^^3^–8.75 × 10^−^^3^) and that estimated for the 42 samples from the UK 2001 outbreak (8.89 × 10^−^^3^, 95%HPD 8.88 × 10^−^^3^–8.91 × 10^−^^3^), when computed by the Student *t*-test (*t* = 15.142, *p* = 0.000). It should be noted that although sample (F) from OB-Shrops came from a healing lesion estimated at the time to be 12 days old at collection, this does not significantly impact the calculated nt substitution rate (9 × 10^−^^2^ according to date of collection and 1 × 10^−^^1^ if calculated with a date 12 days earlier).

## 4 Discussion

Molecular characterization of viral VP1 and FG sequences has been used to analyse FMD outbreaks that occurred from 1966 to 1968 in the UK. Unlike previous studies of FMD epidemics (Cottam et al., 2006, 2008b; Valdazo-Gonzalez et al., 2012), the sampling density (10 samples over 8 months) in this study was too low to attempt to reconstruct viral transmission pathways. However, this retrospective study has provided data to define the genetic relationships between viruses recovered from sporadic outbreaks of FMD and the large epidemic that occurred in the UK during 1966–1968. In accordance with the findings of the Northumberland Report, samples from the OB-Shrops epidemic showed a closer genetic relationship to sequences from contemporary South American isolates compared to all other field samples or cell culture isolates analysed. However, the ability to confidently identify the precise source of virus in this epidemic is restricted by the number and temporal distribution of the samples and isolates available for testing. Therefore, it remains possible that the route of virus introduction that caused the 1967/68 outbreak was via an as yet uncharacterized source in Europe as speculated in the Northumberland Report.

FG sequences can be used to yield further insights into the genetic relatedness of viruses recovered from outbreaks beyond the resolution provided by VP1 sequences. The genetic difference (214–255 nt) observed between the FG sequence from OB-Warks and all other sequences was partially clarified by recombination analysis. Where samples collected from OB-Shrops and OB-Hants showed greatest sequence similarity (between 96 and 100% across the genome) with the South American isolate O_1_/Campos/BRA/58 (AY593819), the single FG sequence from OB-Warks showed three distinct regions of lower nucleotide identity from this serotype O strain. Subsequent re-analysis indicated that these regions shared greatest sequence similarity to a serotype A strain (A_26_/Argentina/66; AY593770), providing evidence of multi-region recombination events between serotype A and O viruses during the evolutionary history of the OB-Warks sequence. Recombination between FMDV field strains has been documented previously (Abdul-Hamid et al., 2011; Jamal et al., 2011) and these data reinforce the importance of recognising these events to limit their potential to bias phylogenetic relationships and timed phylogenies.

The improved resolution of FG sequences enabled the investigation of evolutionary dynamics, across the viral genome, over the timescale of a single outbreak, including nt substitution rate. Although it would have been useful to have included the FG sequence for the actual index case from Bryn Farm (isolate not available), extrapolations from the estimated most likely common ancestor have provided a number of interesting observations. For example, later OB-Shrops samples (I and J) were more closely related (5 nt difference each) to the earliest sample sequenced from this epidemic (A), although they were collected 187 and 217 days later respectively. Genomic sequences from the remaining three late Shrops samples (F–H), also demonstrated characteristics of having been left evolutionary dormant for a period of time (all lying significantly outside the linear accumulation of nt substitution demonstrated by the five earliest OB-Shrops samples and the dataset from the 2001 epidemic in the UK)*.* It can be hypothesized that virus outside a susceptible host does not replicate or incur nt substitutions, and would therefore appear more like the ancestral virus than would be expected given the time that had elapsed. The suggestion was made by the Ministry of Agriculture (Anon, 1969a) that 12/18 cases of FMD that occurred on the same farm twice were due to a recrudescence of the disease, possibly as a result of infected hay remaining on the farm after the original outbreak. However, none of the five late samples from OB-Shrops occurred on farms from which infected samples had previously been collected. Fomite transmission of infected material to previously FMD-free premises, including hay, could also result in this apparent ‘slowing’ of virus evolution during this epidemic, for which these result provide evidence. Furthermore, FMD virus survival outside of a host may have been improved during the winter months of 1967/68. Although improved as compared to VP1 sequencing, the resolution of FG sequencing was insufficient to identify ambiguities indicative of evolutionary intermediates within the samples studied here. Future work could involve the utilisation of next-generation sequencing (Wright et al., 2011; Radford et al., 2012) to investigate the genetic heterogeneity present within individual samples of FMDV.

The equivalent clock rates observed for sequences determined from early OB-Shrops samples (8.73 × 10^−^^3^ nt substitutions/site/year) and the 2001 UK FMD epidemic (8.66 × 10^−^^3^ nt substitutions/site/year), provide evidence for a comparable clock rate across two FMDV topotypes within serotype O, Europe–South America (EURO–SA) and Middle East-South Asia (ME-SA), respectively.

In conclusion, this retrospective study indicates that there were at least three separate incursions of FMD into the UK during the period between 1966 and 1968. These data confirm that earlier UK outbreaks, including those in Northumberland, Hampshire and Warwickshire did not have epidemiological links to the large 1967/68 epidemic. This study reinforces the idea that nucleotide sequences can make an important contribution to understanding the epidemiology of FMD epidemics, and analyses of these data can provide insights that can be used to control and eradicate the disease.

## Figures and Tables

**Table 1:** Details of epithelium samples from which FMDV full genome sequences were derived.

Within outbreak sample ID	Outbreak (OB-)	Outbreak duration	Epi. sample type	Sample collection date	County	World reference laboratory No.	Approximate lesion age (h)	Sequence length (nt)	Total No. of nts sequenced	Average times coverage of each base	GenBank accession No.
–	Hants	6 Jan 1967 to 3 Feb 1967	Bovine tongue	6 Jan 1967	Hants	BFS 1810A	N/A	8177	26393	3.23	JX869177
–	Warks	8 Sep 1967 to 11 Sep 1967	Bovine DP	8 Sep 1967	Warks	BFS 1836	6	8177	45434	5.48	JX869178
A	Shrops	21 Oct 1967 to 6 Jun 1968	Bovine foot	31 Oct 1967	Shrops	BFS 1848	6	8176	35045	4.29	JX869179
B			Bovine foot	14 Nov 1967	Shrops	BFS 1889	12	8176	30647	3.75	JX869180
C			Bovine tongue	8 Dec 1967	Shrops	BFS 1950	6-8	8176	25659	3.14	JX869181
D			Bovine tongue	6 Jan 1968	Heref	BFS 11/68	12	8176	30485	3.73	JX869182
E			Bovine tongue	10 Feb 1968	Staffs	BFS 41/68	8	8173	50125	6.13	JX869183
F			Bovine foot	13 Feb 1968	Ches	BFS 45/68	288	8176	87275	10.68	JX869184
G			Bovine tongue	22 Mar 1968	Ches	BFS 63/68	8	8176	51187	6.26	JX869185
H			Bovine tongue	7 Apr 1968	Ches	BFS 69/68	18	8176	48464	5.84	JX869186
I			Bovine tongue	5 May 1968	Shrops	BFS 86/68	24	8176	54360	6.61	JX869187
J			Ovine^a^	4 Jun 1968	Shrops	BFS 89/68	N/A	8176	44809	5.46	JX869188

N/A not available.

a Epithelium type not available.

**Table 2:** Details of cell culture isolates and one epithelium sample from which FMDV VP1 sequences were derived.

Isolate name	Original material collection date	Location, Country	GenBank accession No.
O_1_/Lombardy/ITL/46^b^	1946	Lombardy, Italy	M58601
O_2_/Flanders/BEL/47^a^	1947	Flanders, Belgium	JX869189
O_2_/Brescia/ITL/47^b^	1947	Brescia, Italy	AY593826
O/M11/MEX/52^a^	1952	Mexico	JX869190
O_1_/Campos/BRA/58^b^	1958	Campos, Brazil	AY593819
O/GRE/1/63^a^	1963	Florida, West Macedonia, Greece	JX869191
O_1_/Brugge/BEL/63^b^	1963	Bruges, Belgium	EU553836
O_1_/Lausanne/SWI/65^b^	1965	Lausanne, Switzerland	M15974
O_1_/Argentina/c.65^b^	c. 1965	Argentina	AY593814
O/BFS 1782/UK/66^a^^,^^c^	22 Jul 1966	Northumberland, UK	KC287135
O_1_/Kaufbeuren/FRG/66^b^	1966	Kaufbeuren, Germany	X00871
O_1_/BFS 1860/UK/67^b^	01 Nov 1967	Wrexham, Cheshire, UK	AY593815
O/FRA/1/81^a^	Mar 1981	Côtes-du-Nord, France	JX869192
O/UKG/15/81^a^	19 Mar 1981	Jersey, Channel Islands, UK	JX869193
O/UKG/16/81^a^	21 Mar 1981	Isle of Wight, UK	JX869194

a VP1 sequence determined for the current analysis.

b VP1 sequence obtained from GenBank.

c VP1 sequence derived from a clinical epithelium sample (OB-Northumb).
